# Evaluation of Computer-Based Cognitive Training on Mild Cognitive Impairment in Parkinson’s Disease (PD-MCI): Α Review

**DOI:** 10.3390/jcm14093001

**Published:** 2025-04-26

**Authors:** Stamatia Kotsimpou, Ioannis Liampas, Metaxia Dastamani, Chrysa Marogianni, Polyxeni Stamati, Antonia Tsika, Lampros Messinis, Grigorios Nasios, Efthimios Dardiotis, Vasileios Siokas

**Affiliations:** 1 Department of Neurology, University Hospital of Larissa, Faculty of Medicine, School of Health Sciences, University of Thessaly, 41100 Larissa, Greece; skotsimpou@uth.gr (S.K.); liampasioannes@gmail.com (I.L.) ; antonellatsi@hotmail.com (A.T.); mdastamani@yahoo.gr (M.D.); c.marogianni@gmail.com (C.M.); tzeni_0@yahoo.gr (P.S.); 2 Laboratory of Cognitive Neuroscience, School of Psychology, Aristotle University of Thessaloniki, 54124 Thessaloniki, Greece; lmessinis@psy.auth.gr; 3 Department of Speech and Language Therapy, School of Health Sciences, University of Ioannina, 45500 Ioannina, Greece; nasios@uoi.gr

**Keywords:** Parkinson’s disease, cognitive dysfunction, computer-based cognitive training

## Abstract

**Background/Objectives:** Mild cognitive impairment in Parkinson’s disease (PD-MCI) affects approximately 20–50% of patients, and it is associated with an increased risk of dementia. Computer-assisted cognitive interventions (CCTs) have been proposed as a promising method of improving cognitive function in these patients. This review aims to (1) demonstrate the effectiveness of computer-based intervention in PD-MCI, and (2) determine the most effective iteration. **Methods:** A review was performed using PubMed, Google Scholar, and ScienceDirect. Full texts of randomized clinical trials (RCTs) involving CCT intervention in PD-MCI and published in English language journals between 2014 and 2024 were included. **Results:** Of the 747 studies identified, 6 studies fulfilled the eligibility criterion for this review. Patients receiving CCTs showed significant improvements in global cognition and executive function, while mood was not significantly affected in most studies. **Conclusions:** CCT improves cognitive functions, particularly memory and executive abilities, but has little effect on mood. Although the results are encouraging, there are potential methodological biases that need to be considered.

## 1. Introduction

Mild cognitive impairment in Parkinson’s disease (PD-MCI) refers to a transitional stage between normal cognition and Parkinson’s disease dementia (PDD), characterized by cognitive decline that exceeds age-related expectations but does not significantly interfere with functional independence. According to the Movement Disorder Society (MDS) Task Force criteria, PD-MCI is diagnosed based on evidence of impairment in one or more cognitive domains—such as attention, executive function, memory, language, or visuospatial abilities—using either Level I (abbreviated) or Level II (comprehensive) neuropsychological testing frameworks [1]. About 20–50% of patients with PD experience PD-MCI) [2] and mild cognitive deficits can be identified in about 15–25% of newly diagnosed patients with PD, and they may be present even before the onset of motor symptoms [3]. Moreover, more severe white matter lesions are associated with poorer cognitive performance in patients with Parkinson’s disease [4]. Individuals with PD-MCI retain autonomy in activities of daily living, which differentiates the condition from dementia. PD-MCI is clinically relevant as it is associated with an elevated risk of progression to PDD [5,6]. Patients with PD-MCI have an annual dementia rate ranging between 9 and 15%, and the incidence is influenced by various clinical and demographic factors—primarily age, disease duration, and disease severity [3]. A comprehensive neuropsychological assessment is essential for the accurate diagnosis of dementia in PD [7]. Recent research has explored potential risk factors and mechanisms involved in PD. One study investigated the rs616147 polymorphism of the myelin-associated oligodendrocyte basic protein (MOBP) gene as a possible risk factor for PD, building on its known association with amyotrophic lateral sclerosis (ALS) [8]. Another study focused on the gut–brain axis hypothesis, suggesting that gut microbiota dysbiosis may trigger inflammation and contribute to PD development [9]. These deficits can significantly impact an individual’s quality of life, daily functioning, and even driving ability [10].

Several pharmacological treatments that have been used for the management of MCI and Alzheimer’s Disease (AD) have also been tested for PD-MCI. However, only rivastigmine has up to date approval with decent results. Furthermore, non-pharmacological treatments are also being explored, such as physical exercise, brain stimulation, and cognitive training, though more research is needed in this area [11].

Multidomain cognitive training using only computer software resulted in measurable improvements in most cognitive domains affected in Parkinson’s disease patients, such as memory, executive function, processing speed, and attention [12]. Computer-based exercises are often more interactive and engaging for users than traditional methods. This helps maintain the interest and participation of patients [13]. Additionally, the difficulty level of the exercises can be adjusted to the individual needs of each user [14]. Digital technologies enable precise tracking of patients’ progress and the delivery of immediate feedback, thereby enhancing the sense of accomplishment and self-esteem [15]. Patients have easy and comfortable access to the exercises, independent of time and place, facilitating the integration of the intervention into their daily lives. In most cases, computerized cognitive training (CCT) programs are more cost-effective compared to traditional face-to-face sessions with healthcare professionals. CCT has emerged as a promising intervention to mitigate cognitive decline in this population. Cognitive intervention entails implementing structured activities to enhance or maintain cognitive functions through repetitive exercises. These exercises improve specific cognitive skills such as memory, attention, language, executive function, and visual–spatial ability.

PD patients have been exposed to many cognitive training schemes. Some of these are RehaCom [16], memory adaptation programs [17], NEUROvitalis [18,19], and Smartbrain Pro [20,21]. Most patients reported significant improvements after the intervention. For instance, patients training with the RehaCom program reported an improvement in executive functions and a reduction in depressive symptoms [16]. The aim of another memory rehabilitation study using paper and pencil exercises was to train attention shifts to facilitate the improvement of executive function [17]. The NEUROvitalis program improves the well-being of PD patients by enhancing their executive and spatial functions [18]. In the end, both SmartBrain Pro and NEUROvitalis had a positive effect on the patient’s cognitive functions while decreasing depression as well as improving the quality of life in patients with Parkinson’s Disease dementia (PDD), respectively [19,20,21]. There have been a few randomized clinical trials (RCTs) examining the effectiveness of CCT in patients with PD-MCI.

There is no sufficient data in the published literature to evaluate the role of CCT on PD-MCI. This study aims to provide a thorough understanding by (a) demonstrating the effectiveness of computer-based intervention in PD-MCI, and (b) determining the most effective intervention.

## 2. Materials and Methods

### 2.1. Literature Search

To identify the most effective intervention program for patients with PD-MCI, a comprehensive review was conducted using PubMed, Google Scholar, and Science Direct. The following search strategy was employed in PubMed: ((((computer cognitive training) OR computerized cognitive training) OR computer cognitive rehabilitation) OR computerized cognitive rehabilitation) AND (((Parkinson’s disease [MeSH Terms]) OR Parkinson) OR parkinsonism) AND (mild cognitive impairment).

The search strategy in Google Scholar included the terms “PD-MCI” and “computerized cognitive training” and “randomized controlled trials” as exact text. To find more recent studies, we applied a filter for the years 2014–2024.

The search strategy in Science Direct included the terms “PD-MCI” and “computer cognitive training” as exact text.

The final literature search in all three databases was conducted on 13 August 2024.

### 2.2. Eligibility Criteria

Inclusion Criteria: Studies meeting the following criteria were included in the present review: (a) classified as RCTs, (b) including participants diagnosed with PD-MCI, (c) investigation of computer-based cognitive training (CT), and (d) provisions of both pre- and post-intervention cognitive data.

Exclusion Criteria: Studies were excluded based on the following criteria: (a) using other study designs (reviews, meta-analyses, observational studies, case studies, etc.), (b) study protocols, (c) being conference abstracts, (e) not being published in English, (f) lacking both pre- and post-intervention cognitive data, (g) assessing different interventions (not CT) or different CT techniques (not computer-based), and (h) including participants with other neurological conditions (e.g., stroke).

Regarding study types, meta-analyses were excluded from this review to ensure a focused analysis of original data derived from primary studies. This decision was made to avoid potential data overlap and duplication, as meta-analyses would likely include the same studies considered in this review. Our aim was to provide a clear, undiluted synthesis of primary-level evidence related to computer-based cognitive training in Parkinson’s disease.

### 2.3. Data Extraction

**Figure 1:**
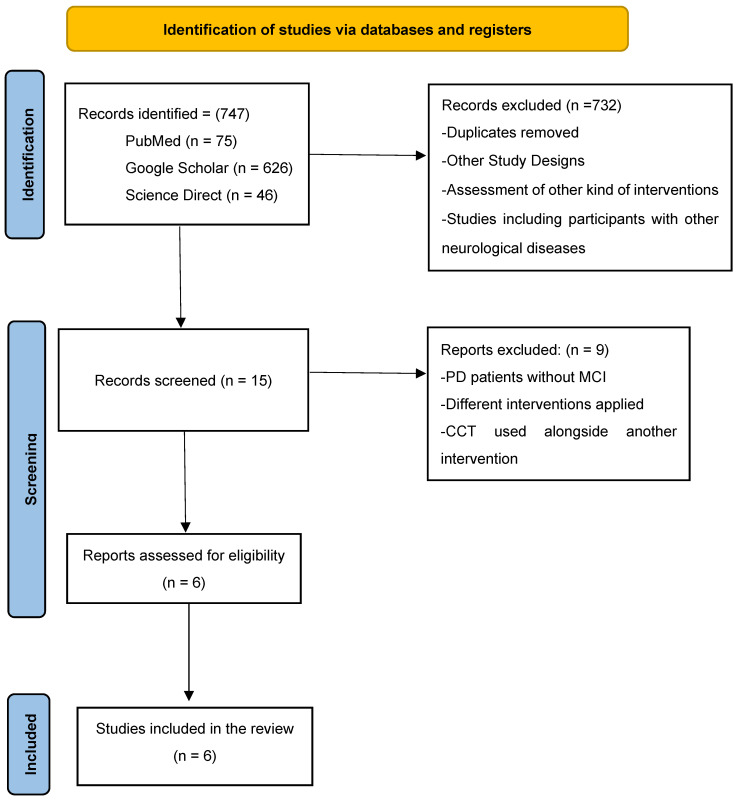
PRISMA 2020 flow diagram [23,24,25,26,27,28].

Retrieved abstracts were carefully evaluated, and full texts were reviewed when inclusion criteria could not be determined. The PRISMA guidelines for reporting systematic reviews were followed [22]. Additional information related to the PRISMA checklist is available in the Supplementary Materials. References from the included papers were also evaluated for eligibility. Full-text screening of potentially relevant articles was performed by two independent reviewers (S.K.) and (I.L.); disagreements were resolved by consensus or with the involvement of a third reviewer (V.S.). Further information regarding the procedure of the selection for eligible studies is presented in Figure 1 [22].

### 2.4. Risk of Bias Assessment Tool

**Figure 2:**
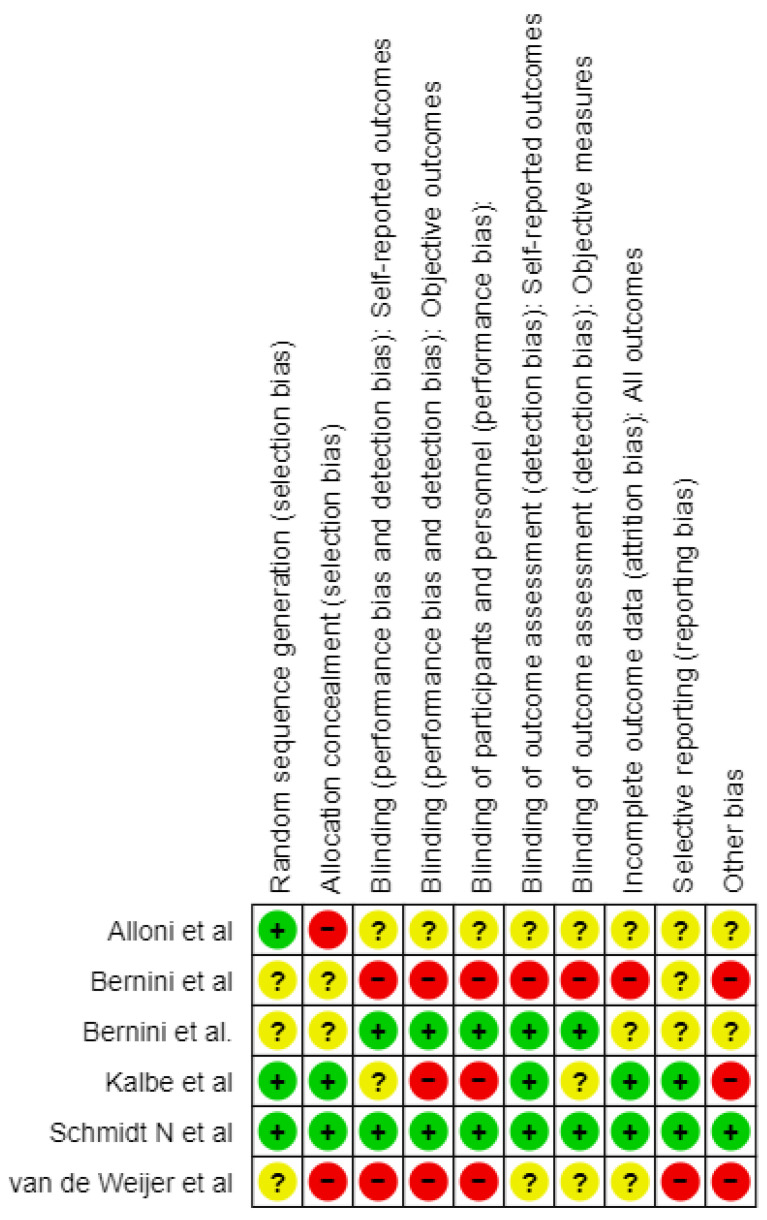
Risk of bias graph [23,24,25,26,27,28].

The risk of bias (RoB) was evaluated using the Cochrane tool for Systematic Reviews of interventions [29]. The following ten methodological aspects were considered: random sequence generation, allocation concealment, blinding of self-reported outcomes, blinding of objective outcomes, blinding of participants and personnel, blinding of outcome assessment for self-reported outcomes, blinding of outcome assessment for objective measures, incomplete outcome data, selective reporting, and other sources of bias. Each aspect was classified as having a low risk of bias (+), unclear risk of bias (?), or high risk of bias (−) based on the methodological features and reporting in the studies that were reviewed (Figure 2).

## 3. Results

### 3.1. Selected Studies

During the study selection, 747 records were identified. This included 75 records from PubMed, with 626 from Google Scholar and 46 supplied by ScienceDirect. Following the evaluation process, 15 records were included in the assessment, whereas 732 were discarded due to ineligible study design, other types of interventions being evaluated, participants having other neurological disorders, or the lack of a control group, along with duplicates. These 15 reports were further evaluated for eligibility, but 9 were excluded because they reported patients with PD without MCI, using unrelated interventions, or combined Edu-Cognitive Therapy with other interventions. In conclusion, six studies fulfilled the eligibility criterion for this review.

According to Alloni et al. (2018) [23], PD-MCI patients with Hoehn and Yahr Scale values of ≤4 were included, while Mini-Mental State Examination (MMSE) and Montreal Cognitive Assessment (MoCA) scores were used to evaluate their cognitive status. The research focused on logical executive functions, category recognition, sequencing, motor speed, attention, focus, and functional planning. The training group (TG) underwent an intervention with the CoRe training program for four weeks, where they improved significantly in various cognitive domains compared to the control group (CG).

Bernini et al. (2019) [24] concentrated on PD-MCI patients meeting the Hoehn and Yahr Scale and Unified Parkinson’s Disease Rating Scale (UPDRS) III, who had MoCA cognitive levels of 24.9 ± 3.5. It sought to compare the impacts of combined cognitive and physical rehabilitation (CoRe) versus conventional physical rehabilitation addressing reasoning–executive functions, memory, and attention/processing speed among others. The intervention group recorded medium to large improvements in executive functions as well as global/general cognitive performance.

Kalbe et al., 2020 [25] involved PD-MCI patients at Hoehn and Yahr Stage 3 or UPDRS Stage IV with a cognitive status of MoCA < 26. This study targeted executive functions such as attention, memory, and visuocognition using a standardized NEUROvitalis program which was delivered twice per week, for a total of 24 weeks, and each session lasted about 90 min. Executive function, phonemic fluency, and physical activity during the intervention session improved.

Van de Weijer et al. (2020) [26] assessed PD-MCI subjects in stage three of the Hoehn and Yahr Scale. The research examined memory, executive functions, attention, working memory, visuospatial processing speed, and psychomotor speed. Participants in TG underwent a web-based CT program (MyCognition AquaSnap) which showed improvements in their executive function and memory compared to the CG.

Bernini et al. (2021) [27] studied patients having scored ≤3 on the Hoehn and Yahr Scale measured by MMSE scores as well as MoCA scores. The study’s primary outcomes were global cognition, executive function, working memory, and attention/processing speed. According to the study’s methodology, the TG received CCT with CoRe software, the extended version, and paper-and-pencil cognitive training or an unstructured intervention (CG). In all groups, the treatment lasted 3 weeks (four individual meetings per week). Standardized neuropsychological measures were evaluated at the beginning of research (T0) and at the end of the treatment period (T1). Compared with the CG, those assigned to TG demonstrated significant improvements in memory as well as executive functions and attention/processing speeds.

Schmidt et al. (2021) [28] included patients recently diagnosed with PD according to the Movement Disorders Society task force level I criteria with a cognitive status of MoCA < 26. The study focused on memory, attention, visuospatial abilities, and psychoeducation. Furthermore, TG participants received the NEUROvitalis program, while CG participants underwent a low-intensity physical therapy intervention. Memory and attention improved more in the TG than in CT.

### 3.2. Information and Main Results per Study

Alloni et al. (2018) [23] did a prospective randomized controlled single-blind clinical trial in Italy to investigate how CT affects the neurocognitive performance measures of PD-MCI patients. A significant difference was observed between MoCA scores that were obtained from the two groups after CT treatment as compared to control ones. The average MoCA score in the CT group (23.5) is higher than that of CG (21.9). However, like the work performed by Kalbe et al. (2020) [25], there were no significant differences regarding change in mood between both groups, while executive functions improved among patients receiving CT. Additional study information is included in Table 1.

### 3.3. Reviewing the Risks of Bias: Comparing Methodologies

While assessing the risk of bias in the studies incorporated in this review, several cross-cutting problems were noticed that may have affected the findings.

Alloni et al. (2018) [23] carried out a study that enrolled 31 patients and employed a randomization that was appropriate considering the even distribution of the baseline demographics. However, even with such a meticulous approach, dropouts are a major problem as many patients do not always complete the study up to the final follow-up. This might potentiate some effects on the overall findings. The study was single-blinded and used objective measures to assess its outcomes; however, the possibility that there is a bias in the reporting results cannot be ruled out and needs careful consideration when evaluating the findings.

Bernini et al. (2019) [24], even though they used a valid method to generate a randomization list with a “random number generator” software, the extended version of CoRe, did not provide information on how they ensured allocation concealment which could introduce bias. Being an open-label RCT, performance bias is possible, especially with respect to subjective outcomes. Although it managed missing data, its lack of blinding poses challenges, especially in cognitive assessments. Additionally, despite efforts to alleviate some concerns, the way in which multiple comparisons are handled in relation to multiple outcomes and time points during this study leaves room for selective reporting.

In the study of Van de Weijer et al. (2020) [26], although the process of randomization was mentioned, there were no specifics about how it was carried out or whether allocation concealment was ensured. The presence of non-adherence to intervention as well as lack of blinding among assessors gives room for potential bias. Although the study used imputation to address missing data, the high dropout rate and the potential impact of missing data on the results are concerning. For subjective outcomes, especially the absence of blinding during outcome assessments creates the risk of bias.

Kalbe et al. (2020) [25] utilized an online program for randomization that was run by a colleague not related to the study, but did not clarify whether allocation concealment was maintained or not. The authors blinded the outcome assessors; however, it is uncertain if participants were aware of their group assignment leading to bias in research findings. The study handled missing data through intention-to-treat analysis, and the dropout rate was low (4.7%). The absence of a published protocol raises concerns about selective reporting, as well as issues with blinding, allocation concealment, and potential selective reporting.

Bernini et al. (2021) [27] randomized patients into three groups, although the allocation process is unclear, which raises concerns regarding selection bias. The study was double-blinded, and the neuropsychologist who assessed the outcomes was unaware of the group allocation. Additionally, there were no changes in medication allowed during the intervention, which minimizes the risk of bias due to deviations from the intended interventions. There were dropouts in the CCT and PCT groups (three and two patients, respectively). The study does not provide information on how missing data were managed. Outcomes were measured by a blinded neuropsychologist using standardized and validated tools, further minimizing the risk of measurement bias. The study mentions multiple outcomes, but it is not clear whether all pre-specified outcomes were reported. This lack of clarity could introduce a risk of reporting only favorable outcomes, resulting in selective reporting bias. The study has some concerns, primarily due to the randomization process and the potential for selective reporting of outcomes.

The Schmidt et al. (2021) [28] study had successful randomization, and its outcome assessors were blinded to reduce selection bias. Medication intake and a controlled environment during interventions curbed deviations in the research. However, the observed high dropout rate was addressed using appropriate statistical methods (24% at 12-month follow-up). In addition, both primary and secondary outcomes were reported exhaustively, and validated assessment tools were used, which makes this study trustworthy although the dropout rate should be carefully considered.

Bernini et al. (2019) [24] carried out an RCT to investigate the effects of CT on neuropsychological measures in PD-MCI patients. They acknowledge that there are significant improvements in the global cognition and executive functions in the CT group of the patients that were reflected by their better performance at MoCA and the Trail Making Test (TMT). The impact of cognitive training on mood was not significant.

The NEUROvitalis program was used on PD-MCI patients by Kalbe et al. (2020) [25]. The study is a multicenter, double-blind RCT. It was found that the TG showed significant improvement in global cognitive function compared to the control group. Since the mean MoCA score was 25.8 in TG as against 24.7 in CG (*p* = 0.03). In addition, executive functions, as assessed by one of the neuropsychological tests like TMT B, significantly influenced the performance, with 68.2 s as the time taken by subjects of the TG compared to 74.5 for CG participants (*p* = 0.04). However, concerning feelings and emotional well-being, no major differences were noticed between the two groups.

Van de Weijer et al. (2020) [26] conducted an RCT in the Netherlands regarding the difference in the effect of CCT over placebo on cognition and mood in patients with PD-MCI. It was shown in the research that the global cognitive function measured by the MoCA test improved significantly in the CT group. Among the TG participants, executive functioning was also increased. Additional effects on mood were not measured.

The study conducted by Bernini et al. (2021) [27], was designed as a double-blind, randomized, controlled trial to investigate the effect of cognitive training on patients with PD-MCI in three arms. The training group had a notable improvement in cognition, especially in terms of verbal fluency, outperforming a CT with 15.4 words, compared to a CG whose average words were 13.8 (*p* = 0.04). It also presented positive changes in global cognition. Even with these cognitive improvements, the research found no significant differences in mood between these groups, which suggests that cognitive training seems to improve specific cognitive functions, but is likely to have little impact on mood in PD patients.

Schmidt et al. (2021) [28] proceeded to investigate the consequences of CT on PD-MCI patients in a multicenter, double-blind RCT conducted in Germany. The findings were that there was a considerable improvement in cognition for the training group, especially the one that related to the overall cognition performance evaluated with the MoCA. There was also an improvement in executive functions for the TG, but no differences were found in the CG. On the Geriatric Depression Scale (GDS), however, there were no pronounced changes in mood with the respective results (5.3 in the TG vs. 5.8 in the CG, *p* = 0.22). Analytical information is shown in Table 2.

## 4. Discussion

To determine the most effective intervention program for patients with PD-MCI, a review was performed on PubMed, Google Scholar, and ScienceDirect databases. The search included the terms “PD-MCI”, “computer cognitive training”, and “randomized controlled trials” for the period of 2014–2024. The risk of bias was assessed based on the Cochrane methodology, analyzing various factors such as randomization, allocation concealment, and participant blinding. In total, after reviewing 747 articles, only 6 studies met the inclusion criteria. The results showed that computerized cognitive training improved patients’ executive functions and memory but had no significant effect on participants’ mood.

Across the studies, patients in training groups showed better cognitive performance compared to the CG. For instance, in the study of Kalbe et al. (2020) [25], the TG had a higher MoCA mean score of 25.8 compared to the CG’s 24.7, with a *p*-value of 0.03, indicating significant improvement because of the intervention. Similar findings are noted in the studies by Alloni et al. (2018) [23] and van de Weijer et al. (2020) [26] where participants in the TG performed much better on tests measuring global cognitive functions in comparison to the TG. There were also improvements in executive functions due to the interventions. For example, in the study by Bernini et al. (2021) [27], the TG performed better than the CG in verbal fluency, with the average number of words produced this time being 15.4, with 13.8 in the CG, with a *p*-value of 0.04. Similarly, in the research conducted by Kalbe et al. (2020) [25], there was a small *p*-value indicating a better performance from the TG in the TMT-B test when compared to CG and the results were as follows 68.2 s and 74.5 s, respectively. Nevertheless, there were no reported effects on mood from these treatments. In most cases, there were no significant differences in terms of mood outcomes between intervention and control groups, as shown in the majority of studies conducted so far; for example, in the study of Schmidt et al. (2021) [28] GDS scores did not differ much among groups (TG = 5.3 vs. CG = 5.8) and had a *p*-value of 0.22 which was non-significant. These findings suggest, therefore, that while these interventions improve cognitive abilities, they have limited impact on mood; hence, this could require different or additional therapeutic approaches.

Although a meta-analysis could potentially provide a more precise quantitative summary of the findings, it was not deemed appropriate for this review due to substantial heterogeneity among the included studies. The selected studies varied considerably in terms of study design, sample characteristics, cognitive training protocols, outcome measures, and reporting formats. This methodological diversity precluded meaningful statistical pooling of the data. Therefore, a narrative synthesis was chosen as the most suitable approach to integrate and interpret the available evidence. This method allowed for a more flexible and context-sensitive comparison of findings while acknowledging the limitations imposed by the heterogeneity of the data.

The review underscores that cognitive training interventions, particularly those focusing on memory, executive functions, and attention, can significantly improve cognitive outcomes in patients with PD-MCI. However, the limited effect on mood indicates a need for supplementary treatments. Despite the promising results, it is important to consider potential biases, such as incomplete blinding, issues with handling missing data, and selective reporting, when interpreting the effectiveness of these interventions.

There are many strengths and limitations of reviewed papers. The risk of false positive results due to multiple outcome measures cannot totally be excluded. Some common methodological issues include lack of allocation concealment reporting, blinding was not carried out well enough, and selective outcome reporting may have been an issue. While many studies displayed robust methodologies and sound statistical analyses, caution was required when interpreting the results because blinding, randomization, and handling of missing data raised concerns. Each one of these studies provides valuable information about how cognitive training can affect PD-MCI, but this also emphasizes some highlighted methodological problems which indicate that potential bias needs to be considered cautiously reviewing these findings.

## 5. Conclusions

Regarding these studies, CT continually demonstrated significant improvements in cognitive functions. It was most effective in global cognition and executive functions in PD-MCI patients. However, it had a minor effect on mood as most studies showed no difference between training groups and control groups. This implies that while CT enhances cognitive abilities, there is little evidence suggesting an impact on mood. Larger sample sizes and fewer methodological limitations in future research may be key to drawing more conclusive remarks about the efficacy of cognitive training.

## Figures and Tables

**Table 1:** Additional information per study.

Study	Country	Single/Multi Training	Double/Single Blind	Parallel Arm	Participants per Group
Alloni et al. (2018) [23]	Italy	Single-Training	Single Blind	YES	TG = 17; CG = 14
Bernini et al. (2019) [24]	Italy	Single-Training	Single Blind	YES	TG = 17; CG = 18
Kalbe et al. (2020) [25]	Germany	Multi-Training	Double Blind	YES	TG = 31; CG = 30
van de Weijer et al. (2020) [26]	Netherlands	Multi-Training	Double Blind	YES	CCT = 21; CG = 20
Bernini et al. (2021) [27]	Italy	Multi-Training	Double Blind	YES	TG1 = 21; TG2 = 14; CG= 18
Schmidt et al. (2021) [28]	Germany	Multi-Training	Double Blind	YES	TG = 28; CG = 26

TG = training group; CG = control group.

**Table 2:** Summary of the procedural characteristics and main findings of the retrieved studies.

Authors	*n*	PD Stages	Cognitive Status	Cognitive Domains Targeted	Technique/Design	Outcome Measures	Duration and Frequency	Results
Alloni et al. (2018) [23]	31	Hoehn and Yahr Scale ≤ 4	MMSE: 25.35 ± 2.60 MOCA: 20.41 ± 3.28	logical-executive functions, category recognition, sequencing and order, auditory-visual integration, memory and association, pattern recognition and logical thinking, attention and focus, functional planning	Training group: CoRe tool. Control group: usual care or standard treatment.	MMSE, MOCA, Verbal Span, Digit Span, CBTT, Logical Memory Test with immediate and delayed recall, Rey’s 15-words test with immediate and delayed recall and RCF-dr, RM47, Weigl’s Sorting test, FAB, FAS, TMTA—TMTB, Stroop tests), RCF-copy	Patients who performed cognitive intervention were subjected to 12 individual sittings, lasting 45 min, using the CoRe system, over 4 weeks (3 sittings/week). Patients in the control group did not perform cognitive training; they only used a sham intervention.	G1: Significant improvements in 12 out of 21 tests, including MoCA, Rey’s 15-word, Logical Memory, Stroop, etc. G2: Minimal improvement in Rey Complex Figure (delayed recall); worsening in Stroop time interference. T0 to T1: G1 showed significant cognitive improvements. G2 showed limited improvement and cognitive worsening in some tasks. T1 to T2: Significant decline observed in G1 (MoCA and FAS), while G2 showed worsening across multiple cognitive tests (MoCA, FAS, MMSE). T0 to T2: G1 maintained improvements in Rey’s 15-word test, Weigl’s Sorting, and Stroop time. G2 showed a general cognitive decline.
Bernini et al. (2019) [24]	35	Hoehn and Yahr Scale and UPDRS III	MoCA: 24.9 ± 3.5	logical-executive functions, attention/processing speed, working memory, and episodic memory	Standard physical rehabilitation plus cognitive intervention with CoRe (intervention group—G1) or standard physical rehabilitation only (control group—G2).	PDQ-8, BDI, MMSE, MoCA, DIGIT SPAN, CBTT, VERBAL SPAN, REY’S 15 WORD TEST-ir, REY’S 15 WORD TEST-dr, LOGICAL MEMORY TEST-ir, LOGICAL MEMORY TEST-dr, RM47, WEIGL’S TEST, FAB, TMT A, TMT B, ATTENTIVE MATRICES, STROOP TEST TIME, STROOP TEST ERROR, PHONOLOGICAL FLUENCY (FAS), SEMANTIC FLUENCY, RCF copy, RCF-dr	CoRe program consisted of 12 individual sessions (3 sessions/week) each lasting 45 minutes of computer-based logical-executive tasks	G1: Medium/large improvements in cognitive performance and executive functions compared to G2. MoCA proved more sensitive than MMSE in detecting improvements. G2: Cognitive decline over 7 months; motor performance improved in both groups, but only G1 showed cognitive improvements.
Kalbe et al. (2020) [25]	61	Hoehn and Yahr Scale ≤5 and UPDRS ≤ IV	MoCA < 26	targeting executive functions, memory, attention, and visuocognition	CT, the standardized NEUROvitalis program CG, a low-intensity physical activity program developed by a sports scientist which aimed to be beneficial for PD patients but to have minimal effects on cognition	The primary study outcomes were memory and executive functions. Secondary outcomes were attention, working memory, visuocognition, language, IADL, self-reported physical activity, depression, QoL, self-experienced attention deficits, and motor impairment including the motor score of the Unified Parkinson’s Disease Rating Scale (UPDRS-III) and freezing of gait (FOG)	Two sessions per week for 90 min over six weeks, conducted in groups of three to five individuals	G1: Improvement in executive functions, phonemic fluency, and a slight improvement in physical activity. G2: Slight improvement in digit span (backward). No significant differences were observed in other neuropsychological or motor symptoms. T0 to T1: Both groups showed slight improvement in some cognitive tasks, but the intervention group (G1) showed more substantial progress.
van de Weijer et al. (2020) [26]	41	Hoehn and Yahr stage ≤ 3	Mild cognitive impairment according to MDS criteria	attention, working memory, episodic memory, psychomotor speed, executive function	Web-based computerized cognitive training ‘health game’ targeting multiple cognitive domains over 12 weeks (via MyCognition AquaSnap). Control group: waiting list.	Neuropsychological assessments, self-report questionnaires, and online cognitive assessment (MyCQTM)	A total of 3 weekly sessions of 30 min each, for at least 12 weeks (primary phase). Participants scheduled their own agenda, and each session duration was not fixed. Both groups could voluntarily play the gamified CT from weeks 12 to 24 (secondary phase). The waiting-list control group did not train in the primary phase but was allowed to train in the secondary phase.	G1: Positive effects in executive function and memory compared to the control group. G2: The control group showed no significant improvements during the primary phase, but similar improvements were observed during the secondary phase after voluntary engagement in cognitive training.
Bernini et al. (2021) [27]	53	UPDRS III/ Hoehn and Yahr scale score ≤ 3	MMSE: 25.01 ± 2.62, MOCA: 19.09 ± 2.84	global cognition, episodic long-term memory, logical-executive functions, working memory, attention/processing speed.	CCT (CoRe), PCT, or CG groups using random numbers.	MMSE, MoCA, Verbal Span, Digit Span, CBTT, Logical Memory Test (immediate and delayed recall), Rey’s 15-word test (immediate and delayed recall), RCF, RM47, Weigl’s Sorting test, FAB, Semantic fluency (animals, fruits, car brands), FAS, Attentive Matrices, TMT A-B, Stroop test, RCF-copy	The intervention lasted 3 weeks with 4 weekly 45 min sessions	G1: Significant improvements in MoCA, global cognition, executive functions, and attention/processing speed, while PCT and CG groups showed no significant changes. G2: No significant cognitive changes observed. T0 to T1: G1 showed significant improvements in multiple cognitive domains, while G2 showed no improvements.
Schmidt Ν et al. (2021) [28]	54	Movement Disorders Society task force Level-II criteria	MoCA < 26	executive function, memory, attention, visuospatial abilities, psychoeducation	G1 received the NEUROvitalis program. G2 received a low-intensity physical therapy program focusing on improving motor function without targeting cognition.	CVLT, ROCFT, Regensburger word fluency tests, Modified card sorting test, BADS, d2-R, WAIS-III, Benton JLO, CERAD, BNT, ACL, Bayer ADL Scale, BDI-II, PASE, PDQ-39, SPAD, UPDRS III, FoGQ	Groups of three to five patients, in two 90 min sessions per week for a total duration of six weeks, were encouraged to continue engaging cognitively and physically after the end of the training phase, without undergoing new sessions until follow-up assessments.	G1: Significant improvements in verbal and nonverbal memory at 6 months, but no significant changes at 12 months. G2: No significant changes in memory or executive function. T0 to T1: Significant interaction effect for memory in the CT group, but no effect on executive function. No significant predictors for memory improvement after CT.

Abbreviations: G1 = intervention group; G2 = control group; ir = immediate recall; dr= delayed recall; M = mean; UPDRS III = Unified Parkinson’s Disease Rating Scale III; PDQ-8 = 8-Item Parkinson’s Disease Questionnaire; BDI = Beck Depression Inventory; ADL = Activities of Daily Living; IADL = Instrumental Activities of Daily Living; MMSE = Mini-Mental State Examination; MoCA = Montreal Overall Cognitive Assessment; CBTT = Corsi’s block-tapping test; RM47 = Raven’s Matrices 1947; FAB = Frontal Assessment Battery; TMT A and B = Trail Making Test parts A and B; RCF = Rey Complex; CVLT: California Verbal Learning Test; ROCFT: Rey–Osterrieth Complex Figure Test; BADS: Behavioural Assessment of the Dysexecutive Syndrome; d2-R: d2-R Test of Attention; WAIS-III: Wechsler Adult Intelligence Scale III; Benton JLO: Benton Judgment of Line Orientation; CERAD: Consortium to Establish a Registry for Alzheimer’s Disease; BNT: Boston Naming Test; ACL: Aphasia Check List; Bayer ADL Scale: Bayer Activities of Daily Living Scale; BDI-II: Beck Depression Inventory II; PASE: Physical Activity Scale for the Elderly; PDQ-39: Parkinson’s Disease Questionnaire 39; SPAD: Self-perceived deficits in attention questionnaire; FoGQ: Freezing of Gait Questionnaire.

## Data Availability

Not applicable.

## Supplementary Materials

The following supporting information can be downloaded at https://www.mdpi.com/article/10.3390/jcm14093001/s1: File S1: PRISMA 2020 checklist.

## Abbreviations

The following abbreviations are used in this manuscript:

ACL	Aphasia Check List
ADL	Activities of Daily Living
ALS	Amyotrophic Lateral Sclerosis
BADS	Behavioural Assessment of the Dysexecutive Syndrome
b-adl scale	Bayer Activities of Daily Living Scale
BDI	Beck Depression Inventory
BDI-II	Beck Depression Inventory II
Benton JLO	Benton Judgment of Line Orientation
BNT	Boston Naming Test
CBTT	Corsi’s block-tapping test
CCT	Computer-Based Cognitive Training
CERAD	Consortium to Establish a Registry for Alzheimer’s Disease
CT	Cognitive Training
CVLT	California Verbal Learning Test
d2-R	d2-R Test of Attention
dr	Delayed Recall
FAB	Frontal Assessment Battery
FoGQ	Freezing of Gait Questionnaire
G1	Intervention Group
G2	Control Group
IADL	Instrumental Activities of Daily Living
ITT	Intention-to-Treat
ir	Immediate recall
M	Mean
MCI	Mild cognitive impairment
MMSE	Mini-Mental State Examination
MoCA	Montreal Overall Cognitive Assessment
MOBP	Myelin-associated Oligodendrocyte Basic Protein
PD	Parkinson’s disease
PASE	Physical Activity Scale for the Elderly
PDQ-39	Parkinson’s Disease Questionnaire 39
PDQ-8	8-Item Parkinson’s Disease Questionnaire
RCF	Rey Complex Figure
RCT	Randomized Controlled Trials
RM47	Raven’s Matrices 1947
ROCFT	Rey-Osterrieth Complex Figure Test
SPAD	Self-perceived deficits in attention questionnaire
TMT A and B	Trail Making Test parts A and B
UPDRS III	Unified Parkinson’s Disease Rating Scale III
WAIS-III	Wechsler Adult Intelligence Scale III
